# Ionic Liquids Incorporating Polyamide 6: Miscibility and Physical Properties

**DOI:** 10.3390/polym10050562

**Published:** 2018-05-22

**Authors:** Xin Zheng, Qingqing Lin, Pan Jiang, Yongjin Li, Jingye Li

**Affiliations:** 1 CAS Center for Excellence on TMSR Energy System, Shanghai Institute of Applied Physics, Chinese Academy of Sciences, No. 2019, Jialuo Road, Jiading District, Shanghai 201800, China; myhero521@126.com; 2 College of Material, Chemistry and Chemical Engineering, Hangzhou Normal University, No. 16 Xuelin Rd., Hangzhou 310036, China; lqq0770709@163.com (Q.L.); jiangpan0629@gmail.com (P.J.); 3 University of Chinese Academy of Sciences, Beijing 100049, China

**Keywords:** PA6, ionic liquid, homogenous blends, physical connection, dynamic rheological behaviors

## Abstract

The effects of 1-vinyl-3-butyl imidazole chloride (VBIM) on the structure and properties of Polyamide 6 (PA6) were investigated systematically. It was found that PA6/VBIM blends were homogeneous without phase separation. The glass transition temperature (*T*_g_) of PA6 increased with small VBIM loadings followed by the decreasing in *T*_g_ with further increasing the amount of VBIM. The crystallization temperature decreased with the addition of VBIM because of the strong interactions between VBIM and the PA6 matrix, as well as the dilution effect when large amounts of VBIM was introduced to the matrix. According to rheological testing, small amounts of VBIM enhanced the storage modulus and melt viscosity of PA6. Tensile tests also show an increase in strength and modulus at relatively low loadings of VBIM. The strength of PA6 with only 1 wt % VBIM improved by 108% compared to that of neat PA6. Fourier transform infrared (FTIR) investigations revealed that the ions of VBIM preferred to form hydrogen bonds with amide groups in PA6. Therefore, VBIM acts as physical connection point for the neighboring PA6 molecular chains. The specific interactions between VBIM and PA6 account not only for the enhanced melt viscosity of PA6, but also for the improved mechanical properties. Moreover, outstanding antistatic property was also observed. The surface resistivity of the sample with 1 wt % VBIM was 1.50 × 10^10^ Ω/sq, which means good electric dissipation property.

## 1. Introduction

Polyamide 6 is an important engineering plastic [1,2,3]. One of the main features of PA6 is that the lattice of PA6 is formed by holding the well-defined hydrogen bonded sheets together. The hydrogen bonding is generated between polar amide groups on adjacent molecular chains [4]. The existence of hydrogen bonding endows PA6 with excellent physical properties, such as outstanding mechanical properties, high thermostability, good resistance to mild acid or alkali, and so on. Despite of the superior comprehensive properties, specific modification or functionalization of PA6 were still in urgent demand. Generally, blending PA6 with another polymer was often used to prepare high-performance PA6 composites. Fernandes and his coworkers [5] prepared reinforced PA6 composites with cellulose fiber, the composites exhibited good interactions between components and notably high mechanical properties. Additionally, PA6 nanocomposite, with nanofillers dispersed in PA6 matrix, is an important category of modified PA6 materials which exhibited improved properties, such as mechanical, dimensional, and barrier properties, thermal stability, and flame retardant property [6,7,8]. Damm [9] also reported the preparation of PA6/silver nanocomposites with long-term antimicrobial property. In addition, functionalized fabric is one of the most important applications of PA6. According to Pang [10], PA6/TiO_2_/PANI composite nanofibers were fabricated using electrospinning, electrospraying, and an in situ chemical polymerization method. The prepared nanofibers show excellent gas sensing property and can be used as gas sensors, potentially. 

On the other hand, room temperature ionic liquids (RTILs) are unique salts which are fluids at temperatures around or below 100 °C [11]. They are composed of only ions, usually including a bulky, asymmetrical organic cation and an anion (organic or inorganic) [12,13]. Due to the unique ion structure, RTILs usually exhibit excellent properties, such as negligible vapor pressure, high thermostability, and a wide range of solubility and miscibility; thus, they have been widely used as green chemistry solvents [14,15,16,17]. Moreover, RTILs also show outstanding ionic conductivity [15,18,19,20], thermal conductivity [21,22,23], antimicrobial property [24,25,26], lubricity [27,28] and so forth. The multifuntionality of RTILs makes them versatile modifier for polymer. Our group has previously investigated the cooperation of RTIL to different polymer matrices. One of the authors [29] prepared polypropylene (PP)/glass fiber (GF) composites with excellent antistatic performance by blending with a small amount of ionic liquid, in which GF networks offered an orbit for ions to transfer. In another work, antistatic nanofibers based on poly (vinylidene fluoride) (PVDF) and a RTIL, 1-butyl-3-methylimidazolium hexafluorophosphate [BMIM][PF_6_], were fabricated via electrospinning [30]. Yu and his coworkers [25] reported a kind of antisuperbug material by grafting a RTIL (1-butyl-3-vinyl imidazole chloride [BVIM][Cl]) onto the surface of cotton fabric via irradiation induced polymerization method. By now, few works were published to report the modification of PA6 by ionic liquid [31,32]. 

In this work, an ionic liquid named 1-vinyl-3-butyl imidazole chloride [VBIM][Cl] (VBIM for short, hereafter) was used to fabricate PA6/VBIM blends by simple melt-mixing. VBIM was chosen because the vinyl groups in IL make it possible to undergo further reactions in the system, which will be reported in the next work in the near future. The incorporation of ionic liquid into the PA6 matrix was assumed to introduce excellent comprehensive functionalities of RTILs in PA6. The effects of VBIM on the structure and properties of the PA6 matrix were investigated systematically.

## 2. Materials and Methods

### 2.1. Materials

Polyamide 6 (PA6) with commercial name UBE Nylon 1022B (density 1.14 g/cm^3^, melting temperature 215–225 °C) was purchased from UBE Industries, Ltd. (Tokyo, Japan). 1-vinyl-3-butyl imidazole chloride [VBIM][Cl] (*M*_m_ = 187 g/mol) was produced by Lanzhou Yulu Fine Chemical Co., Ltd., Lanzhou, China and used as received. 

### 2.2. Preparation of the PA6/VBIM Blends

Before melt mixing, polyamide 6 pellets and VBIM were dried at 110 and 80 °C in a vacuum oven overnight, respectively. The PA6/VBIM blends were prepared using a Haake Polylab QC mixer (Thermo Fisher, Waltham, MA, USA) with a twin screw. In particular, PA6 and ionic liquid were sheared at a rotation speed of 20 rpm at 220 °C for 2 min, followed by 50 rpm for 5 min. 

After melt mixing, all samples were hot-pressed at 230 °C under 10 MPa pressure for 3 min, then cooled at the same pressure to films with thicknesses of 500 μm. The films were directly used for the following characterizations. The dumbbell-shaped specimens for the tensile tests were prepared by injection molding according to ISO 527-2-5A.

### 2.3. Characterization

Oscillatory rheological characterizations were performed on a physical rheometer (MCR301, Anton Paar Instrument, Graz, Austria). The diameters of the parallel plates are 25 mm, the gap between the two plates is 1 mm. The dynamic frequency sweep experiments of all samples were carried out at 235 °C under a dry nitrogen atmosphere to prevent thermo-oxidative degradation of the PA6 matrix. The frequencies used in this system ranged from 0.1 to 100 rad/s. The strain amplitude was set to be 1%.

Differential scanning calorimeter (DSC, TA-Q2000, TA Instruments, New Castle, PA, USA) measurements were used to track the crystallization and melting behaviors of all samples under a high-purity nitrogen atmosphere. All samples were heated to 250 °C and held for 5 min to erase the thermal history, then cooled to 20 °C, followed by heating again to 250 °C. Both the cooling and heating rate were 10 °C/min. The first cooling and second heating curves were recorded.

Dynamic mechanical analysis (DMA, TA-Q800, TA Instruments, New Castle, PA, USA) was carried out with the multi-frequency strain mode from 30 to 150 °C. The heating rate is 3 °C/min. The dynamic loss (tan δ) and storage modulus (*G*′) were determined at a frequency of 5 Hz.

Thermogravimetric analysis (TGA, TA-Q500, TA Instruments, New Castle, PA, USA) was applied to estimate the thermostability of all samples. The samples were heated from room temperature to 650 °C with a heating rate of 10 °C/min, during which a high-purity nitrogen atmosphere was blown continuously to remove the oxygen from the furnace.

An X-ray diffractometer (XRD, Bruker-D8, Bruker, Karlsruhe, Germany) was used to detect the crystalline forms of the samples. The data was collected from 5° to 40° at a scanning speed of 1°/min. The step interval is 0.02°.

The Fourier transform infrared (FTIR) spectroscopy measurements were conducted on film samples with a transmittance mode using a FTIR spectrometer (Bruker Vertex 70V, Bruker, Karlsruhe, Germany). The FTIR spectra were recorded from 4000 to 400 cm^−1^ at a resolution of 2 cm^−1^, and 64 scans were averaged.

The microstructure of the fractured surface of samples was observed using a field emission scanning electron microscope (SEM, Hitachi S-4800, HITACHI, Tokyo, Japan). The samples were fractured in liquid nitrogen followed by coating with gold before observation. An acceleration voltage of 3 kV was used for sample observation.

Electrical conductivity was measured on an ultrahigh resistivity meter (MCP-HT450) (Mitsubishi, Nagasaki, Japan). The URS probe electrode was adopted and the testing voltage was 10 V. The thickness of the samples was about 500 μm.

Mechanical properties were carried out on an Instron universal material testing system (model 5966) (Instron, Norwood, MA, USA) at room temperature. The tensile speed is 5 mm/min. 

## 3. Results

### 3.1. The Morphology of PA6 and the PA6/VBIM Blends

**Figure 1:**
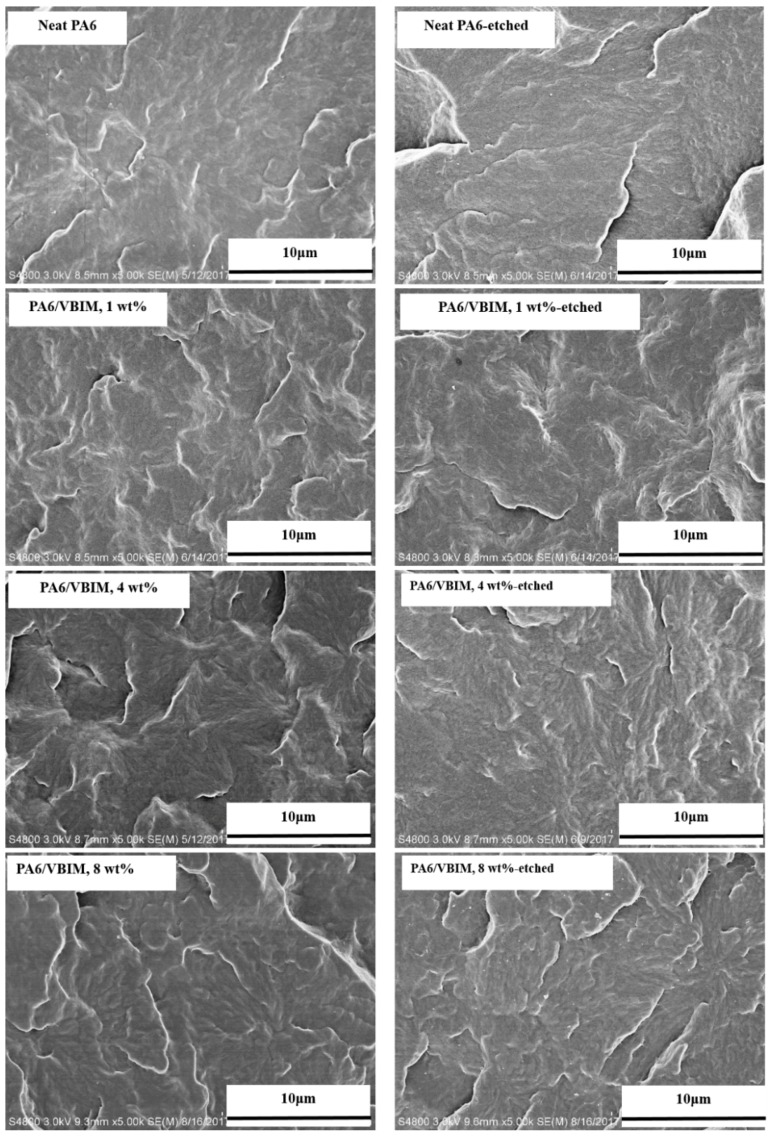
SEM images of the cross-fracture surface of PA6 and PA6/VBIM blends with different VBIM loadings before and after etching.

Figure 1 shows the SEM images of the fracture surfaces of PA6 and PA6/VBIM blends with different VBIM loadings (taking 1, 4, 8 wt % as examples). It is obvious that neat PA6 shows a homogenous phase. With the addition of VBIM (1–8 wt %), no apparent phase separation is observed. To verify the location of VBIM in PA6 matrix, the cross-sections were etched with methanol, which is a good solvent for VBIM, and then observed using SEM. It is clear from the images that the microstructures of etched samples are almost the same with those before etching. This means that VBIM shows good miscibility with PA6 matrix. Additionally, a qualitative elemental mapping distribution of VBIM-incorporated PA6 was conducted and shown in Figure S1. It is found that VBIM dispersed homogenously in the PA6 matrix, which again confirmed the good miscibility of VBIM in PA6.

### 3.2. The Crystallization Behaviors of PA and the PA6/VBIM Blends

#### 3.2.1. The Crystalline Form

**Figure 2:**
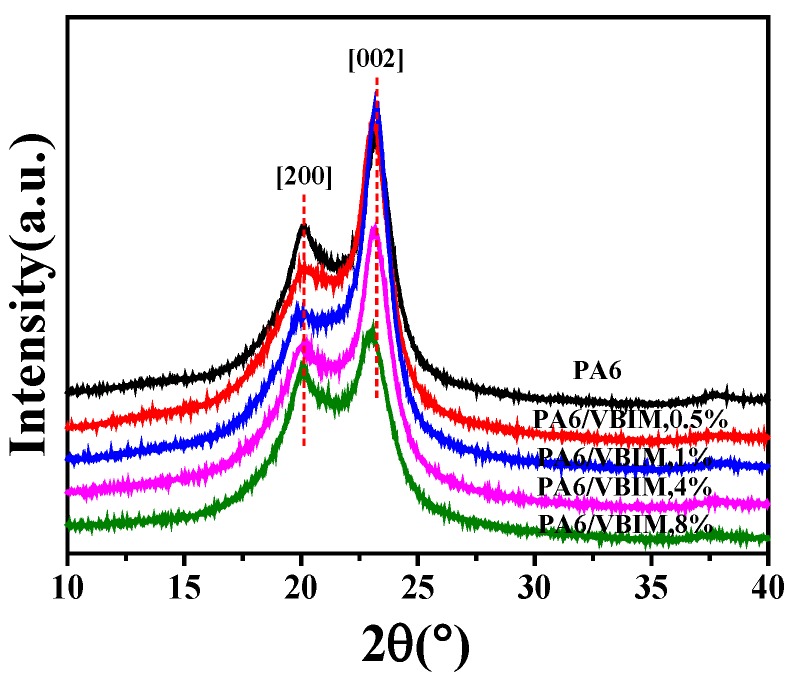
XRD patterns of neat PA6 and the PA6/VBIM blends.

Many crystalline polymorphs can be identified in PA6, of which *α* and *γ* are two major crystal forms [33]. The chains in *α* crystals are the fully extended zigzag conformation, and the hydrogen bonds are among antiparallel chains; while in *γ* crystals, the hydrogen bonds are formed among parallel chains with twisted conformation, which lead to shorter chain-axis repeat and the formation of pleated sheets [4,34,35]. The crystalline forms of neat PA6 and the PA6/VBIM blends were characterized by XRD, as shown in Figure 2. Only one typical crystalline form can be distinctively identified from the pattern of PA6: two peaks at 2θ = 20.1°, 2θ = 23.2° are assigned to the [200] and [002] reflections of *α* form crystals, respectively [36]. The crystalline form does not change with the incorporation of VBIM, implying that, even though there are strong interactions between PA6 and VBIM because VBIM and PA6 are miscible, VBIM does not participate in, and has no effect on the crystallization process. Additionally, the diffraction peaks of the PA6/VBIM, 8 wt % blend shift to a lower position slightly. We consider that the insignificant shift of the peak is caused by the different flatness or thickness of the 8 wt % content blend. Thus, the strong interactions between VBIM and the PA6 matrix may obstruct the process of crystallization, but the VBIM is finally expelled from the crystalline region to the amorphous region so that the crystalline form and interplanar space remain unchanged.

#### 3.2.2. The Crystallization and Melting Behaviors

**Figure 3:**
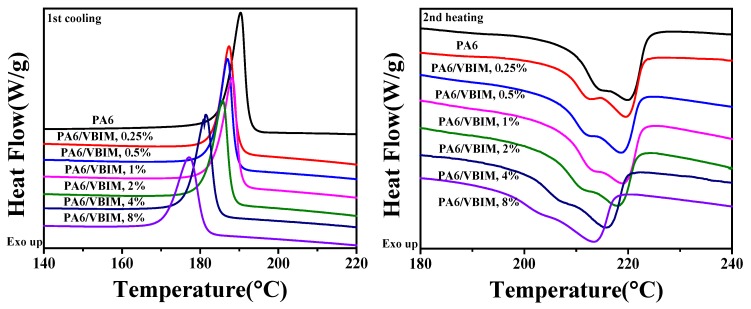
DSC first cooling (after 5 min isothermal for erasing heat history) and second heating curves of PA6 and the PA6/VBIM blends with different VBIM loadings.

The first cooling and second heating traces of neat PA6 and its blends were recorded as shown in Figure 3. The detailed thermal properties, including the melting temperature, melting enthalpy, crystallization temperature, crystallization enthalpy, and the crystallinity are tabulated in Table 1. The crystallinity of PA6 in all samples ($\chi_{c}$) is determined by Equation (1) below:

(1) $$\chi_{c}=\frac{\Delta H_{m}}{\phi \times \Delta H_{m}^{0}}\times 100\%$$

where $\Delta H_{m}^{0}$ is the theoretical melting enthalpy of 100% crystalline PA6 with a value of 190 J/g [37], ϕ is the weight fraction of the matrix in the blends. 

The DSC curves of neat PA6 show a crystallization temperature (*T*_c_) of 190.4 °C and a corresponding melting temperature (*T*_m_) of 219.8 °C in the non-isothermal crystallization process. Generally speaking, the *T*_m_ of polymers is related to the thickness of lamella, crystal form, regularity, and so on. Thus, the apparent shoulder in the melting curve of PA6 may be due to crystals with different regularity or the melting of crystals which were generated during the melting process. It is noteworthy that crystallization and melting behaviors of PA6 change significantly with the combination of VBIM. Specifically, *T*_c_ and *T*_m_ decrease with increasing VBIM loadings in PA6. According to SEM observations and XRD results, it is clear that PA6 and VBIM blends show good miscibility, though there are certainly strong interactions between VBIM and the PA6 matrix, the existence of VBIM does not change the crystalline form and interplanar space of PA6 matrix. As we assumed before, the strong interactions impede the motion of PA6 molecular chains and inhibit the crystallization behavior during cooling process from the melt so that the *T*_c_ decreased. The $\chi_{c}$ of the PA6/VBIM blends with small amounts of VBIM (0.25–1 wt %) do not change much, while they decrease drastically with 2–8 wt % VBIM, which may be attributed to the dilution effect of large amounts of VBIM on the concentration of PA6 molecular chains, as well as the plasticizing effect of VBIM. Additionally, the shoulder is still observed in the melting curve of PA6/VBIM blends, suggesting that VBIM do not participate in the crystallization process, which is in good accordance with XRD patterns. The DSC curves of neat VBIM can be found in Figure S2.

### 3.3. Thermal Behaviors of PA6 and the PA6/VBIM Blends

#### 3.3.1. The Glass Transition Temperature

**Figure 4:**
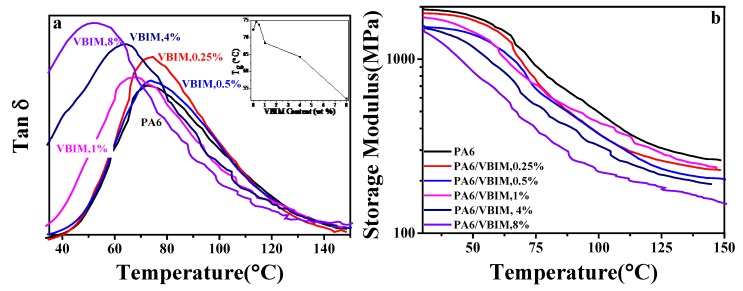
The loss tangent (**a**) and storage modulus (**b**) as functions of temperature for neat PA6 and the PA6/VBIM blends. The inserted sketch on the top right corner in Figure 4a shows how *T*_g_ varies according to VBIM contents in the blends.

Generally speaking, the miscibility of blends can be determined by the glass transition behaviors of the blends. If there was only one *T*_g_ in a binary blend, and the value of *T*_g_ fitted the Fox equation [38] or Gardon-Taylor equation [39] well, then it is deemed that the two components are miscible. The dynamic mechanical analysis (DMA) was used to study the *T*_g_ of neat PA6 and the PA6/VBIM blends. Figure 4 shows the curves of the loss tangent (tanδ) and storage modulus as functions of temperature with a frequency of 5 Hz. The inserted sketch of Figure 4a on the top right corner shows how *T*_g_ varies according to VBIM contents in the PA6 blends. It is found that *T*_g_ initially increased slightly from 72 °C for neat PA6 to about 74 °C for samples with 0.25 wt % and 0.5 wt % VBIM and then decreased drastically to about 52 °C with 8 wt % VBIM. The increase in *T*_g_ indicates that there are strong interactions between VBIM and PA6 matrix that impede the mobility of PA6 chain segment; while the decrease of the *T*_g_ value is due to the plasticizing effect of VBIM. The results are in good accordance with the previous analysis. One may notice that the plasticizing effect of VBIM happened at a relative lower VBIM content (1 wt %) according to DMA results compared to DSC, in which the VBIM started to take plasticizing effect at 2 wt % VBIM loadings. This is mainly because the *T*_g_ was determined by mobility of chain segment in amorphous region of PA6/VBIM blends, and the amorphous region was concentrated with VBIM which was expelled out from crystalline region according to XRD results; while in DSC measurements, the crystallization process was affected by VBIM which was diluted and dispersed uniformly in the melt, thus, a higher content of VBIM will be needed to show plasticizing effect compared to crystallized samples in DMA measurement. From Figure 4b, we can see that the more VBIM was incorporated, the more the storage modulus was decreased. This may be because of the plasticizing effect of the ionic liquid on the PA6 matrix.

#### 3.3.2. Thermal Stability of PA6 and PA6/VBIM Blends

**Figure 5:**
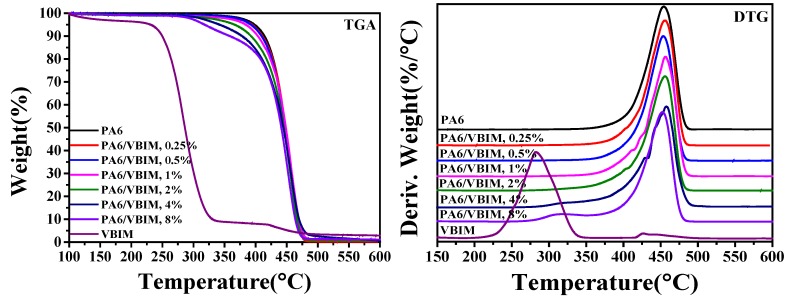
The TGA and DTG curves of neat PA6 and the PA6/VBIM blends.

TGA was used to measure the effect of VBIM on thermal stability of PA6 matrix. TGA and DTG curves are given in Figure 5. It is obvious that VBIM shows low thermal stability with the initial degradation temperature (*T*_5%_) of 231.7 °C. Note that the initial degradation temperature is higher than the processing temperature (220 °C). The *T*_5%_ of neat PA6 is much higher than that of VBIM, and it takes one step to degrade. With the incorporation of VBIM, the degradation with two separated steps is observed from the TGA curves. Obviously, it is VBIM that firstly loses weight at relative lower temperature followed by the degradation of PA6 matrix at higher temperature. The degradation of VBIM is almost negligible in DTG curves when the amount of VBIM is less than 4 wt %, which indicates the homogenous phase of the blends. This confirms again the good miscibility between PA6 matrix and VBIM. An observable VBIM degradation curve appears in blends with 4 wt % and 8 wt % VBIM, and the weight loss percentage of VBIM is in good accordance with the amount of VBIM that we added. Additionally, it is noteworthy that the thermal stability of VBIM increases greatly in the blends, which is ascribed to the strong interactions between PA6 and VBIM. The corresponding parameters of the TG analysis are summarized in Table 2. 

### 3.4. Rheological Properties of PA6 and the PA6/VBIM Blends

The dynamic rheological response is believed to be an effective method for providing information regarding the structure/morphology of materials under small amplitude oscillatory shear (SAOS), during which the morphological variation of blends can be neglected [40]. The rheological properties of multi-component polymers are governed by three main factors: the intrinsic properties of the components; the compositions; and the interfacial interaction between the components [41]. The changes of rheological properties are generally ascribed to mechanical coupling between the components, or interface morphological structure [40].

**Figure 6:**
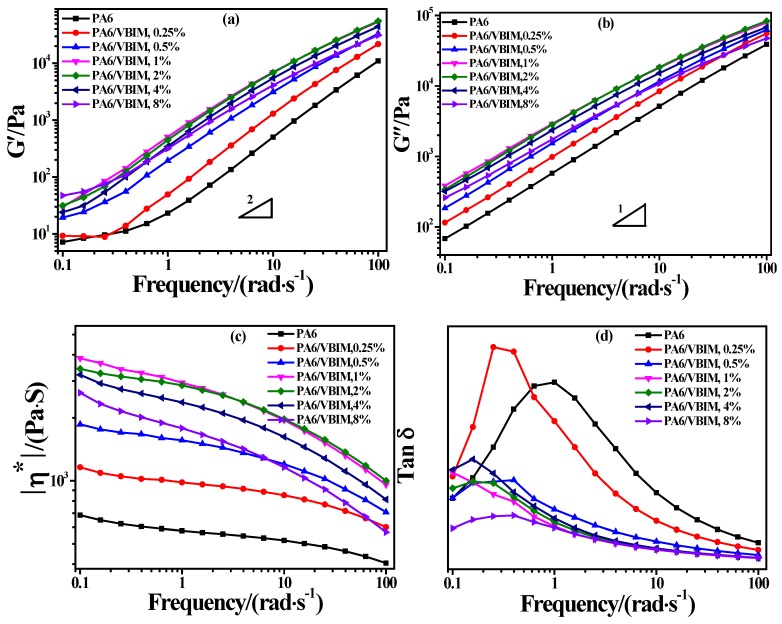

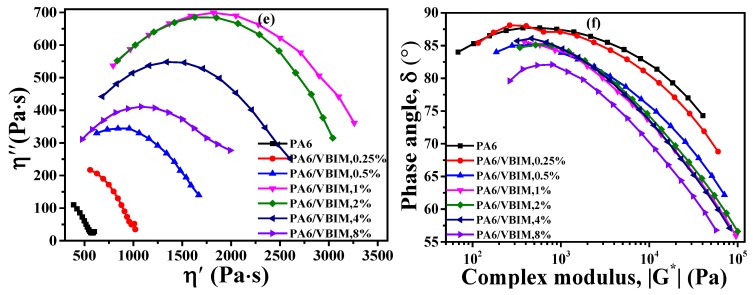
Linear viscoelastic properties from SAOS experiments: (**a**) storage modulus *G*′; (**b**) loss modulus *G*″; (**c**) complex viscosity |η*|; (**d**) damping factor tan δ as functions of angular frequency; (**e**) Cole-Cole plots; and (**f**) vGP plots.

The dynamic rheological behaviors of neat PA6 and PA6 blends with different amounts of VBIM incorporated were characterized using SAOS tests. The dynamic storage modulus *G*′, loss modulus *G*″, complex viscosity |η^*^|, and damping factor Tan δ versus the angular frequency (ω) of PA6 and various PA6/VBIM blends are plotted in Figure 6a–d separately. For neat PA6, the molecular chains are expected to be fully relaxed above the melting temperature so that terminal behavior with relations of *G*′-ω^2^ and *G*″-ω^1^ at low frequencies (terminal region) is predictable according to linear viscoelastic theory [42]. While it can be seen from Figure 6a that the *G*′ curve of PA6 showed a deviation from linearity with a terminal zone slope much smaller than expected at very low frequencies, indicating heterogeneous behavior, which is usually ascribed to the polydispersed molecular weight, phase separation, formation of the network structure, and deformation of droplest in different systems [43,44,45,46,47]. Additionally, the derivation at relative low *G*′ value is sometimes simply ascribed to the instrumental error. The deviation from the monodispersed behavior of PA6 is less obvious in Figure 6b, which means that *G*′ is more sensitive to structural change than *G*″ in the frequency dispersion curves. It is notable that both *G*′ and *G*″ increase with the incorporation of VBIM in the whole range of the testing frequencies compared to those of neat PA6, and reach a maximum in the PA6/VBIM, 1 wt % blend, thereafter decreasing slightly with further increasing VBIM content (2–8 wt %). Furthermore, like neat PA6, all samples with the incorporation of VBIM also exhibit derivation from linearity with a higher modulus plateau. These results indicate that there are stronger intermolecular interactions in PA6/VBIM blends than in neat PA6 even at a molten state [41]. In other words, the interactions between two VBIM molecules or between VBIM and PA6 molecules are stronger than that between PA6 and PA6 molecules. Generally, ionic liquid is used as a lubricant or plasticizer, leading to a decrease in the viscosity or storage modulus of the ionic liquid-incorporated polymer blend. Thus, it is the interaction between VBIM and PA6 molecules that is responsible for the enhanced *G*′, *G*′′ and modulus plateau in PA6/VBIM blends compared to those of neat PA6. Since no significant second-plateau in *G*′ and *G*″ curves is observed, we believe that there is no crosslinking network, but only a physical “connection” (most likely hydrogen bonds in this work) in the blends and it is the physical “connection” that accounts for the enhancement of *G*′ and *G*″. Due to the polarity of amide groups in PA6, we assume that the physical “connection” refers to the interactions between VBIM and amide groups on PA6 molecular chains. The veracity of this claim will be clarified in detail by Fourier infrared transform spectra in the Discussion section. Consequently, the entanglement of molecular chains is enhanced and the motion of PA6 molecular chains is retarded significantly with only small amount of VBIM. This result explains the restrained crystallization behavior well, as we stated previously. To sum up, it is the physical “connection” that accounts for the enhancement of *G*′ and *G*″ in VBIM-incorporated samples. The value of *G*′ and *G*″ reach their peak at 1 wt % VBIM content may be explained by the maximum number of the physical “connection” in PA6/VBIM, 1 wt % sample; while the decrease in the values may due to the plasticization effect or destruction of original hydrogen bond in PA6 offered by a large amount of VBIM (2–8 wt % in this work).

The complex viscosity curves of neat PA6 and PA6/VBIM blends are shown in Figure 6c. It can be seen that |η^*^| increases with a small amount of VBIM (0.25–1 wt %), then declines at higher VBIM content (2–8 wt %), which is in good accordance with the changes of *G*′ and *G*″. However, the value of |η^*^| for all PA6/VBIM blends is still higher than that of neat PA6, particularly at low frequencies. This is due to the fact that the physical interactions between PA6 and VBIM restrict the mobility of PA6 molecular chains, and these kinds of interactions are more stable than those of intra- and inter-chain interactions (mainly hydrogen bonds) which are inherently presented in the PA6 matrix even at temperatures higher than the melting temperature. Additionally, |η^*^| of both PA6 and the PA6/VBIM blends show a dependence of frequency in the whole testing range, especially at very low frequencies, corresponding to non-Newtonian fluid behavior [48]. At high frequencies, the viscosity decreases drastically, indicating a phenomenon of the gel structure destruction and shearing-thinning behavior [49]. Moreover, the shearing-thinning region of the PA6/VBIM blends is shifted to lower frequencies compared to PA6, and the shift is even more notable with the increase of VBIM. According to Malmberg [50], the shear thinning behavior of the polymer with long chain branching differed from that of linear polymer with similar molecular weight. As assumed previously, VBIM is acting as physical “connection” between PA6 chains, the bonded PA6 and VBIM are analogous to long chain branched molecules. It is the “long chain branch”-like molecules, but not the crosslinking network, take responsibility for the change in the shear-thinning phenomenon.

Figure 6d exhibits tan δ curves of PA6 and PA6/VBIM blends as a function of angular frequency. It is believed that tanδ against angular frequency curves are more sensitive to the irreversible structure change of polymers compared to plots of the modulus against angular frequency [41]. For neat PA6, the curve culminates in vicinity of 1 rad/s, which is the typical non-terminal behavior of solid-like polymers. The result is consistent with the conclusions mentioned above. As comparisons, the curves of PA6/VBIM blends culminate at relatively lower frequencies, indicating stronger intermolecular interactions and greater resistance of molecules movement due to the physical interactions between PA6 and VBIM. Furthermore, tan δ decreases remarkably with the addition of VBIM at higher frequencies, and the more the VBIM is incorporated, the lower the value that tan δ drops to, which means an increase of the elastic response of PA6/VBIM blends, as well as an increase in the terminal relaxation time.

The Cole-Cole plot is a curve of imaginary viscosity (η′) versus real viscosity (η″), in which the ω dependence was eliminated. By illustrating characteristic relaxation behavior of polymer systems, the Cole-Cole plot has been identified as an important criterion for the detection of phase separation of two phase systems, like polymer blends and polymer nanocomposites [40,51]. Usually, one smooth semicircle shape of Cole-Cole plot will suggest one kind of long relaxation process, indicating good compatibility between the two phases of polymer systems, or at least good dispersion of one phase in the other one [49]. As is shown in Figure 6e, the Cole-Cole plot of neat PA6 is fitted well to a semicircle, a slight deviation was observed on the right-hand side of the arc because of light chemical crosslinking during melt mixing process as mentioned before. With the incorporation of VBIM, there is only one arc in various PA6/VBIM blends, demonstrating a homogenous system. This claim is in line with morphology observation in Figure 1 in the previous section. The radius of the arc increases at first and reaches a maximum at 1 wt % VBIM content, afterwards decreasing from PA6/VBIM, 2 wt % to 8 wt % blends. The radius of PA6/VBIM, 8 wt % is still larger than that of neat PA6, which is in good accordance with previous analysis. The phenomenon indicates longer relaxation time in the VBIM modified PA6 systems. It is generally accepted that higher molecular weight and stronger intermolecular interactions would bring about longer relaxation time. In this work, a small amount of VBIM acts as physical “connection” points between two PA6 molecular chains so that the modulus and viscosity are enhanced, leading to a longer relaxation time. However, there is no crosslinking network formed based on weak physical interactions, thus, no apparent deviation from the semicircle is observed in PA6/VBIM blends. The number of physical “connection” is rising with increasing VBIM contents in the range of 0.25 wt % to 1 wt %, leading to an enlarged radius of Cole-Cole plots; while excess VBIM (2 to 8 wt %) acts as a plasticizer and facilitates the motion of PA6 molecular chains so that the radius diminishes correspondingly. 

The van Gurp-Palmen (vGP) plot displays the relationship between the phase angle (δ) and logarithmic complex modulus (|G^*^|), which is chosen to verify the time temperature superposition principle, polydispersity of the linear polymer, long chain branched polymer, rheological percolation of polymer blends, and the miscibility of polymer blends [52,53,54,55,56]. The phase angle (δ) is a characteristic response to liquid-like or solid-like behavior and ranges from 90° for ideal Newtonian liquid to 0° for ideal Hookean solid [57]. The vGP plots of PA6 and PA6/VBIM blends are given in Figure 6f. For neat PA6, the δ is close to 90°, corresponding to complete viscosity in the terminal region. The combination of VBIM in the PA6 matrix does not alter the development of the vGP plot compared to that of PA6, which means there is no phase separation in PA6/VBIM blends. However, the vGP plots of PA6/VBIM blends do not merge into the common curve with PA6, and the δ decrease markedly at whole range of |G^*^|. We preferred to attribute the phenomenon to the increased elasticity of PA6 after introducing VBIM due to improved interactions between PA6 and VBIM. 

### 3.5. Physical Properties of PA6 and the PA6/VBIM Blends

#### 3.5.1. Mechanical Properties

**Figure 7:**
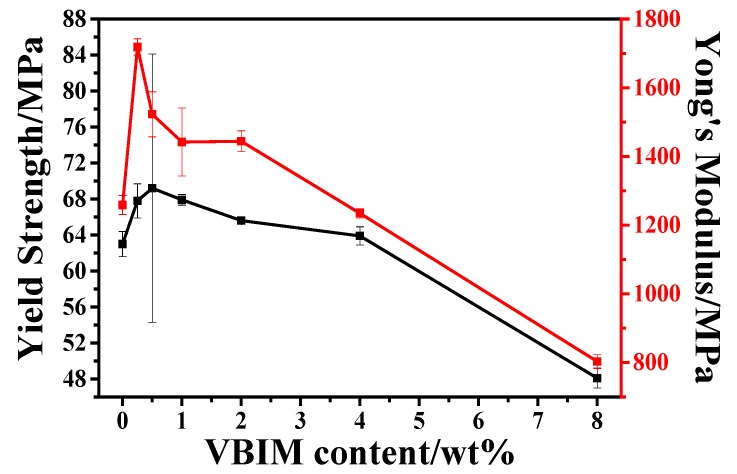
The yield strength and Young’s modulus as functions of the VBIM content.

Tensile tests were carried out on an Instron universal material testing instrument at 25 °C with a tensile speed of 5 mm/min. The corresponding tensile properties of neat PA6 and PA6/VBIM blends are shown in Figure 7. Neat PA6 shows typical high strength (63.0 MPa) and high modulus (1258.7 MPa) tensile behavior. With small amount of VBIM (0.25–2 wt %), both the yield strength and modulus of PA6 blends get enhanced apparently. For instance, the yield strength of PA6/VBIM, 0.5% is 69.2 MPa, which is 110% that of PA6, and the modulus is 20% higher than neat PA6. In consideration of DSC and DMA results, the $\chi_{c}$ of PA6/VBIM, 0.5% is almost the same with neat PA6, but *T*_g_ increases because of formation of physical “connection” and enhanced intermolecular interactions. Thus, it is reasonable to deduce that it is the physical “connection” reinforced intermolecular interactions that account for the improvement of yield strength and modulus in PA6/VBIM blends. Just like the variation of *T*_g_ in dependence of VBIM content, the yield strength and modulus of PA6 blends decrease gradually from 1 wt % to 8 wt %. However, the value of yield strength and modulus with 1–2 wt % VBIM incorporated blends is still higher than that of neat PA6 due to existence of physical “connection”, while blends with 4–8 wt % VBIM show falling yield strength and modulus because of much lower crystallinity compared to neat PA6 and plasticization effect of VBIM at large loadings. The detailed datasheet of elongation and strength at break, as well as the yield strength and Yong’s modulus are concluded in Table S1. 

#### 3.5.2. The Antistatic Performance of PA6 and the PA6/VBIM Blends

**Figure 8:**
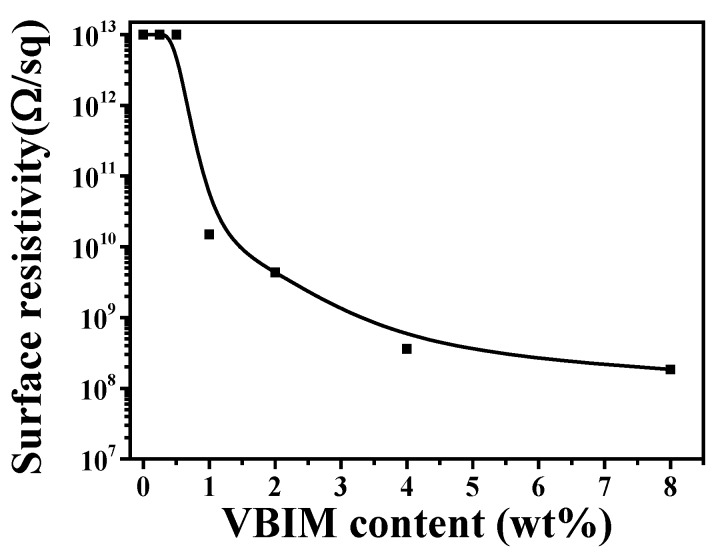
Surface resistivity of PA6 and the PA6/VBIM blends as a function of VBIM contents. This section may be divided by subheadings. It should provide a concise and precise description of the experimental results, their interpretation, as well as the experimental conclusions that can be drawn.

It is well known that the static electric dissipation is especially important for PA6 in industry. Figure 8 shows the electrical conductivity of PA6 and the PA6/VBIM blends. It is clear that VBIM shows a great impact on the antistatic performance of PA6 matrix. It is easy to be found that neat PA6 exhibits insulative characteristic, the value of surface resistivity is too large to be measured under the experimental conditions, which is greater than 10^13^ Ω/sq. No drastic enhancement is observed for the antistatic performance of PA6 matrix with less than 1 wt % of VBIM. The value of the surface resistivity decreases dramatically to 1.50 × 10^10^ Ω/sq with 1 wt % VBIM, which indicates good electric dissipation property of the blends. Usually, an antistatic material is determined by surface resistivity that is lower than 10^12^ Ω/sq. That is to say, we fabricated an antistatic PA6 material with only 1 wt % VBIM, the mechanical properties of which were also improved according to results of tensile tests. Additionally, the antistatic performance would get further enhanced with more VBIM incorporated. In all samples we prepared in this work, no excess VBIM bleeding during melt mixing process and no VBIM migration to the surface of PA6/VBIM films during aging at room temperature for weeks are observed. This again implies the good miscibility between VBIM and PA6. Obviously, the VBIM incorporated PA6 blends exhibit great potential applications for the permanent antistatic engineering plastics.

## 4. Discussion

Ionic liquid, as mentioned in introduction section, was often used as a lubricant, plasticizer, and so on [41,42,58]. It is expected that the combination of the polymer matrix and ionic liquid would bring about decreased viscosity, *T*_g_, $\chi_{c}$, strength, and modulus. We surprisingly find that the |η^*^|, *G*′, *G*″ increased significantly with only small amount of VBIM added to the PA6 matrix in the melt state. Additionally, *T*_c_, *T*_g_, and even the mechanical properties also show interesting changes with the combination of VBIM. The phenomenon reveals the miscibility and apparent intermolecular interactions between PA6 and VBIM. Detailed interaction mechanism is investigated by temperature variable Fourier infrared transform spectra.

**Figure 9:**
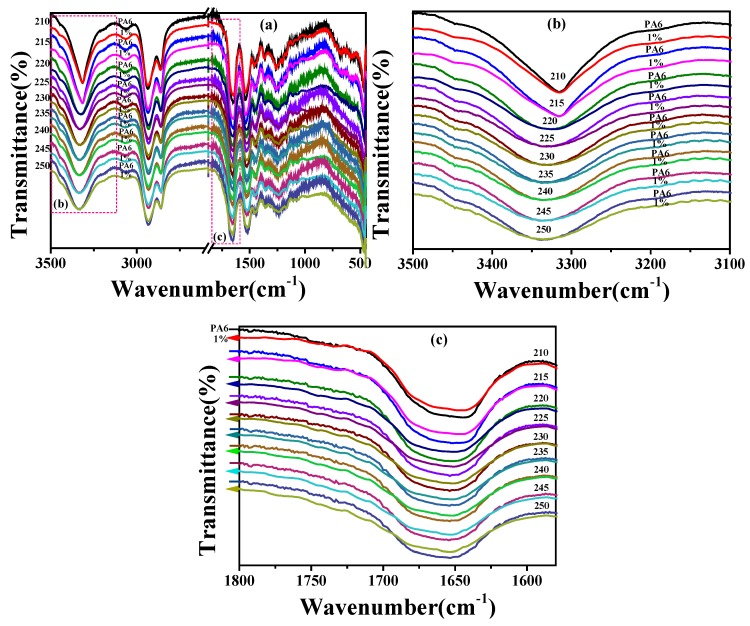
The temperature variable FTIR spectra from 210 to 250 °C of: (**a**) neat PA6 and the PA6/VBIM, 1 wt % blend; (**b**) zoomed area of the N–H stretching band; and (**c**) zoomed area of the amide I band.

The temperature variable FTIR of PA6 and the PA6/VBIM, 1 wt % blend was conducted from room temperature to above the melting temperature at a heating rate of 20 °C/min in order to figure out the variation of hydrogen bond with the addition of VBIM. PA6/VBIM, 1 wt % blend was chosen as an example because of the most significant rheological behavior changes at molten state. The spectra of PA6 and PA6/VBIM, 1 wt % around the melting temperature were recorded and shown in Figure 9. The spectra were plotted to make a comparison between neat PA6 and the PA6/VBIM, 1 wt % blend at the same testing temperature. Since the characteristic peaks of CH_2_ are largely unaffected by hydrogen bond, only N–H stretching and amide I band are focused on and discussed in this part. From Figure 9a we found that the N–H stretching peak of both PA6 and the PA6/VBIM, 1 wt % blend shifts to a higher wavenumber as temperature increasing, besides, the breadth of N–H stretching mode increases from 210 to 250 °C in both systems (see Figure S3). Particularly, the breadth broadened drastically from 215 to 220 °C, as we can see in Figure 9b. The temperature range, according to DSC results, is exactly the melting temperature of semicrystalline PA6 and the PA6/VBIM, 1 wt % blend. Thus it is reasonable to deduce that the hydrogen bonded N–H groups in the crystalline region contribute to the characteristic peak of the N–H stretching vibration before melting. The breadth of PA6/VBIM, 1 wt % is broader compared to neat PA6, which may be related to different contributions for each sample. The small shoulder above 3400 cm^−1^ appeared in all samples is assigned to free N–H. We can conclude that there are several contributions to N–H peak in both PA6 and PA6/VBIM, 1 wt % blend, the combination of VBIM is likely to change the species and positions of each contribution to the peak. The relevant curve fitting analysis will be discussed later in the text. As given in Figure 9c, almost the same tendency as N–H stretching mode is found in amide I band, but the breadth of amide I band increase steadily without abrupt change around melting temperature. Additionally, the characteristic peak of C=O is not a single Gaussian curve in both PA6 and VBIM-incorporated PA6 blends, which is also attributed to several contributions to the peak [59]. 

**Figure 10:**
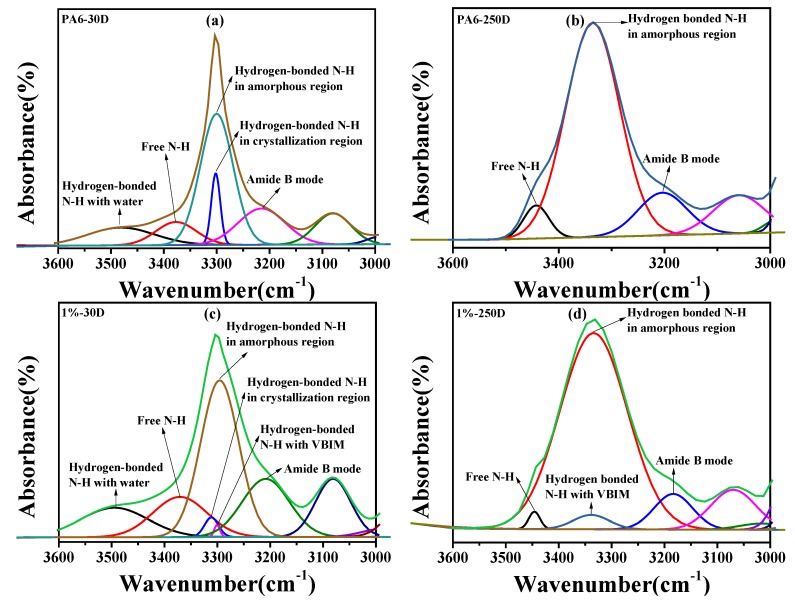
Curve-fitting of the N–H stretching band of (**a**) PA6 at room temperature; (**b**) PA6 at 250 °C; (**c**) PA6/VBIM, 1 wt % blend at room temperature; and (**d**) the PA6/VBIM, 1 wt % blend at 250 °C.

As we mentioned before, there are several contributions to the characteristic peaks (especially N–H and C=O peaks) of PA6 and the PA6/VBIM, 1 wt % blend. As stated by Skrovanek [59], the distinct spectral of C=O groups in amide I band can be divided into three subpeaks from high to low wavenumbers, which are assigned to non-hydrogen-bonded C=O groups, hydrogen bonded C=O groups in the amorphous region, and hydrogen-bonded C=O groups in the crystalline region, successively. In our system, it is difficult to determine a baseline to fit the curve of C=O. However, according to our previous analysis of the N–H stretching band, we have successfully divided the N–H peak of PA6 and PA6/VBIM, 1 wt % at both room temperature and molten temperature. The detailed curve-fitting diagrams are exhibited in Figure 10. We have to point out that, the absolute area of each subpeak makes no sense in temperature variation FTIR because of the shifty absorption coefficient [59]. Therefore, the curve fitting treatment is only conducted for qualitative analysis. For neat PA6, there are five subpeaks which are pointed out and assigned, respectively, in Figure 10a at room temperature. The free N–H in PA6 at room temperature correlates to the existence of water. Beyond the melting temperature, the hydrated hydrogen bonded N–H and hydrogen-bonded N–H in the crystalline region disappear and the shoulder of free N–H is more obvious in PA6, which can be seen in Figure 10b. The generation of free N–H above melting temperature relates to the breakage of the hydrogen bond while, in Figure 10c, a new subpeak emerged at a lower wavenumber than the original hydrogen-bonded N–H in the PA6/VBIM, 1 wt % blend, indicating a stronger interaction happened with the incorporation of VBIM at room temperature. This confirmed our assumption that the chloride anion would coordinate with N–H to form stronger hydrogen bonds than the original ones. The hydrated hydrogen bonded N–H and hydrogen bonded N–H in the crystalline region also vanished with temperature increasing to above melting temperature, and the shoulder of free N–H became obvious under a molten state, as shown in Figure 10d. More importantly, the relative area of free N–H gets smaller and the relative area of new hydrogen-bonded N–H with a chloride anion (as mentioned before) turns out to be larger compared to those in the same blend at room temperature. Firstly, it verifies our assumption that VBIM tend to form new hydrogen bond with free amide groups so that the relative area of free N–H decreased; secondly, the result implies a high thermal stability of the hydrogen bond between amides and VBIM; thirdly, it explains why the viscosity and modulus increased even under molten state. The FTIR spectra at room temperature of PA6 and the PA6 blends with different VBIM loadings are also given in Figure S4. 

**Figure 11:**
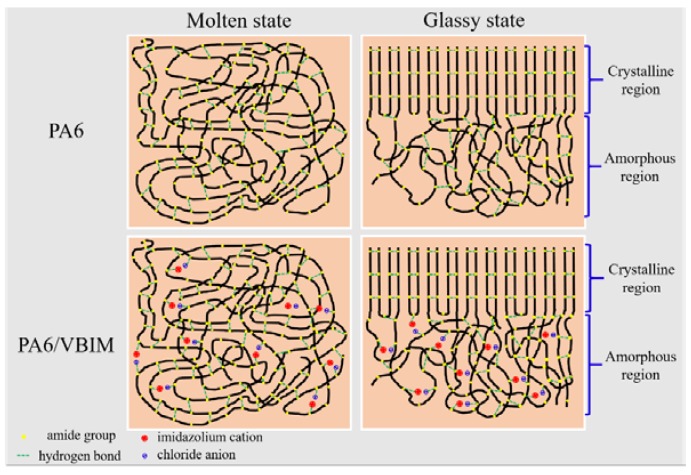
A schematic of the interactions between PA6 and VBIM under molten and glassy states.

**Figure 12:**
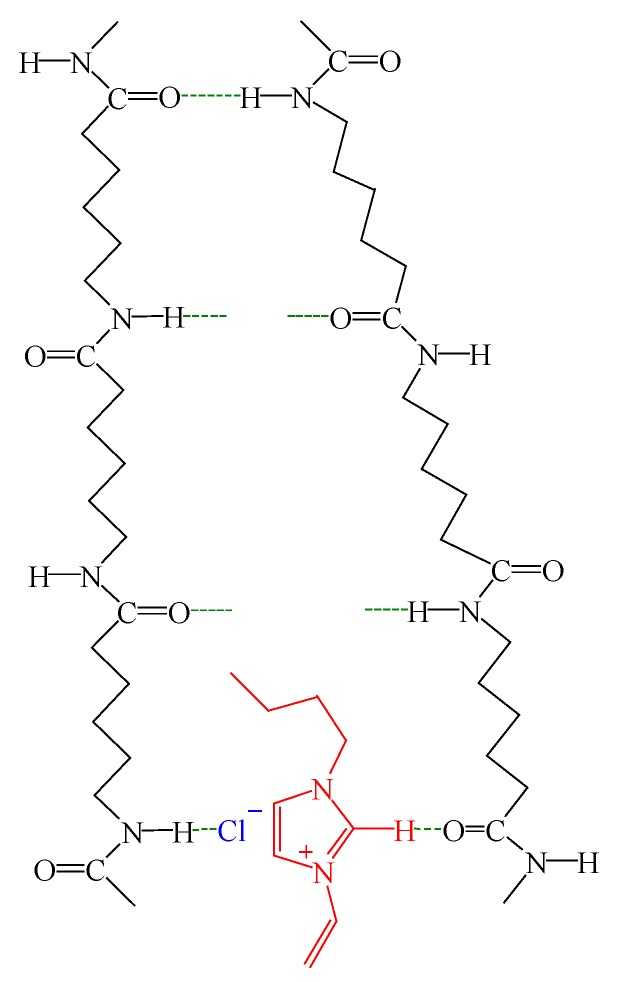
Schematic diagram of destruction and re-building of the hydrogen bond with the incorporation of excess VBIM.

Taking the FTIR results above into consideration, the effects of VBIM on structure and properties of PA6 are well-understood. A schematic diagram is drawn to illustrate the effects of VBIM on PA6 matrix (Figure 11). Under the molten state, there are free amide groups generated because of the breakage of the original hydrogen bond in neat PA6, but most of the amide groups are still hydrogen bonded. The crystallization by cooling down from the melt leads to the formation of *α* crystal which is stable and confirmed by XRD patterns while, in the PA6/VBIM blend, the process will be discussed in two cases according to the content of VBIM. In the first case, the free amide groups in PA6 matrix are occupied by ions with 0.25–1 wt % VBIM at molten state. The more the VBIM is incorporated to PA6 matrix, the more free amide groups are consumed. Almost all free amide groups are taken up with 1 wt % VBIM. To be specific, the imidazolium cations coordinate with the C=O groups in one amide group while the chloride anions interact with N–H groups on another adjacent amide group. In addition to the interaction between ions and amide groups, there are also strong interactions between cations and anions. Consequently, the combination of 0.25–1 wt % VBIM enhanced the interchain interactions of PA6 matrix, leading to good miscibility of VBIM in the PA6 matrix and a prominent enhancement in |η^*^|, *G*′, *G*″, and so on. The value of |η^*^|, *G*′, and *G*″ culminate at 1 wt % VBIM content because almost all free amide groups are re-bonded at this circumstance with the strongest interchain interactions provided. The crystallization process from the melt will be retarded due to strong interactions between VBIM and PA6 molecular chains, resulting in decreased *T*_c_. Based on the results of XRD, VBIM was expelled out from the crystalline to the amorphous region; thus, the concentration of VBIM in amorphous region below melting temperature is higher than that in totally amorphous region. Correspondingly, *T*_g_ increased with 0.25–0.5 wt % VBIM compared to that of PA6, and reached the maximum value at 0.25 wt % VBIM content according to DMA results, which is much less than 1 wt %. In the second case, only a portion of 2–8 wt % VBIM will form hydrogen bond with free amide groups, at the same time, ions of redundant VBIM will interrupt the original hydrogen bond and form a new one with the “freed” amide groups. The destruction of original hydrogen bond and re-building of the hydrogen bond with ions in VBIM are sketched in Figure 12. Hence, the structure and properties of PA6 matrix are determined jointly by both the destruction of original hydrogen bond and the formation of new ones. However, the formation of one new hydrogen bond would sacrifice several original ones. Thus, even though there are stronger interactions between VBIM and PA6, |η^*^|, *G*′, and *G*″ declined slightly with 2–8 wt % VBIM incorporated compared to the PA6/VBIM, 1 wt % blend. Additionally, the crystallization process from the melt will be retarded further, resulting in decreased *T*_c_ and *χ*_c_. The same trend is also observed for *T*_g_.

## 5. Conclusions

In this work, ionic liquid-incorporated PA6 blends were prepared by a simple melt-mixing method and the effects of ionic liquids on the structure and properties of PA6 were studied systematically. Small amounts of VBIM induced an enhanced storage modulus and viscosity of PA6 due to the strong specific interactions. The detailed information about the interactions was revealed by FTIR. To be specific, the chloride anion would form a hydrogen bond with the N–H group, while the imidazolium cation preferred to coordinate with C=O. Taking steric hindrance effect into consideration, it is conceivable that the two ions of one VBIM molecule tends to interact with C=O and N–H from two amide groups on neighboring PA6 molecular chains. It is believed that VBIM is prone to form new hydrogen bonds with free amide groups in PA6. After almost all free ones are occupied, the stronger interactions between VBIM and amide groups, compared to those between amide groups in neat PA6, would break the original hydrogen bond between the amide groups and form new ones. In this case, it should be pointed out that one new hydrogen bond between VBIM and an amide group is at the expense of several original ones because of the bulky size of the imidazolium cation. The strong interactions between VBIM and PA6 endow PA6/VBIM blends with excellent mechanical properties, besides, the incorporation of VBIM also makes PA6 an antistatic material due to the conductivity property of the ionic liquid.

## Figures and Tables

**Table 1:** The detailed thermal properties of PA6 and the PA6 blends with different VBIM contents based on DSC curves.

Sample	*T*_m_/°C	∆*H*_m_/J·g^−1^	*T*_c_/°C	∆*H*_c_/J·g^−1^	$\chi_{c}$/%
Neat PA6	219.8	48.6	190.4	53.1	25.9
PA6/VBIM, 0.25%	219.4	49.0	187.4	48.0	26.1
PA6/VBIM, 0.5%	218.3	49.4	187.1	48.6	26.1
PA6/VBIM, 1%	218.5	49.1	188.2	47.6	25.9
PA6/VBIM, 2%	217.5	44.3	185.8	47.1	23.1
PA6/VBIM, 4%	215.2	44.0	181.5	51.4	22.5
PA6/VBIM, 8%	212.9	37.6	177.3	50.1	18.5

**Table 2:** TGA parameters of PA6 and the PA6/VBIM blends.

Sample	*T*_5%_/°C	*T*_50%_/°C	*T*_max_/°C
PA6	398.0	448.3	454.2
PA6/VBIM, 0.25%	393.3	448.3	455.8
PA6/VBIM, 0.5%	392.5	447.0	453.4
PA6/VBIM, 1%	384.1	448.7	456.4
PA6/VBIM, 2%	366.1	445.4	456.0
PA6/VBIM, 4%	341.0	444.2	457.6
PA6/VBIM, 8%	317.4	441.0	452.4
VBIM	231.7	286.0	282.6

## Supplementary Materials

The following are available online at http://www.mdpi.com/2073-4360/10/5/562/s1, Figure S1. Elemental mapping distribution of PA6, PA6/VBIM, 4 wt % blend (labelled as 4%) and PA6/VBIM, 8 wt % blend (labelled as 8%); Figure S2. The DSC curves of neat VBIM; Figure S3. The plot of N–H width at half height in IR as a function of temperature; Figure S4. FTIR spectra of: (a) neat PA6, VBIM, and PA6/VBIM blends; (b) zoomed area of the N–H stretching band; and (c) zoomed area of the amide I band; Table S1. Basic tensile properties of the neat PA6 and PA6/VBIM blends.
