# Supplementary material for: Ionic Liquids Incorporating Polyamide 6: Miscibility and Physical Properties
Source: Polymers (Basel). 2018 May 22;10(5):562. doi: 10.3390/polym10050562 (PMC6415449; doi:10.3390/polym10050562)
Supplement: Supplementary file 1 [file polymers-10-00562-s001.pdf]

Supporting information for

Ionic Liquids Incorporating Polyamide 6: Miscibility and Physical Properties

Xin Zheng, [1, 2, 3] *Qingqing Lin,* [2] *Pan Jiang,* [2] *Yongjin Li,* [2*] *Jingye Li,* [1*]

[1] CAS Center for Excellence on TMSR Energy System, Shanghai Institute of Applied Physics, Chinese Academy of Sciences, No.2019, Jialuo Road, Jiading District, Shanghai 201800, People's Republic of China; myhero521@126.com

[2] College of Material, Chemistry and Chemical Engineering, Hangzhou Normal University, No. 16 Xuelin Rd., Hangzhou 310036, People's Republic of China; lqq0770709@163.com (Q. L.); jiangpan0629@gmail.com (P. L.)

[3] University of Chinese Academy of Sciences, Beijing 100049, People's Republic of China

* Correspondence: yongjin-li@hznu.edu.cn (Y. L.); lijingye@sinap.ac.cn (J. L.)

Tel.: +86-0571-2886-7026 (Y.L.)

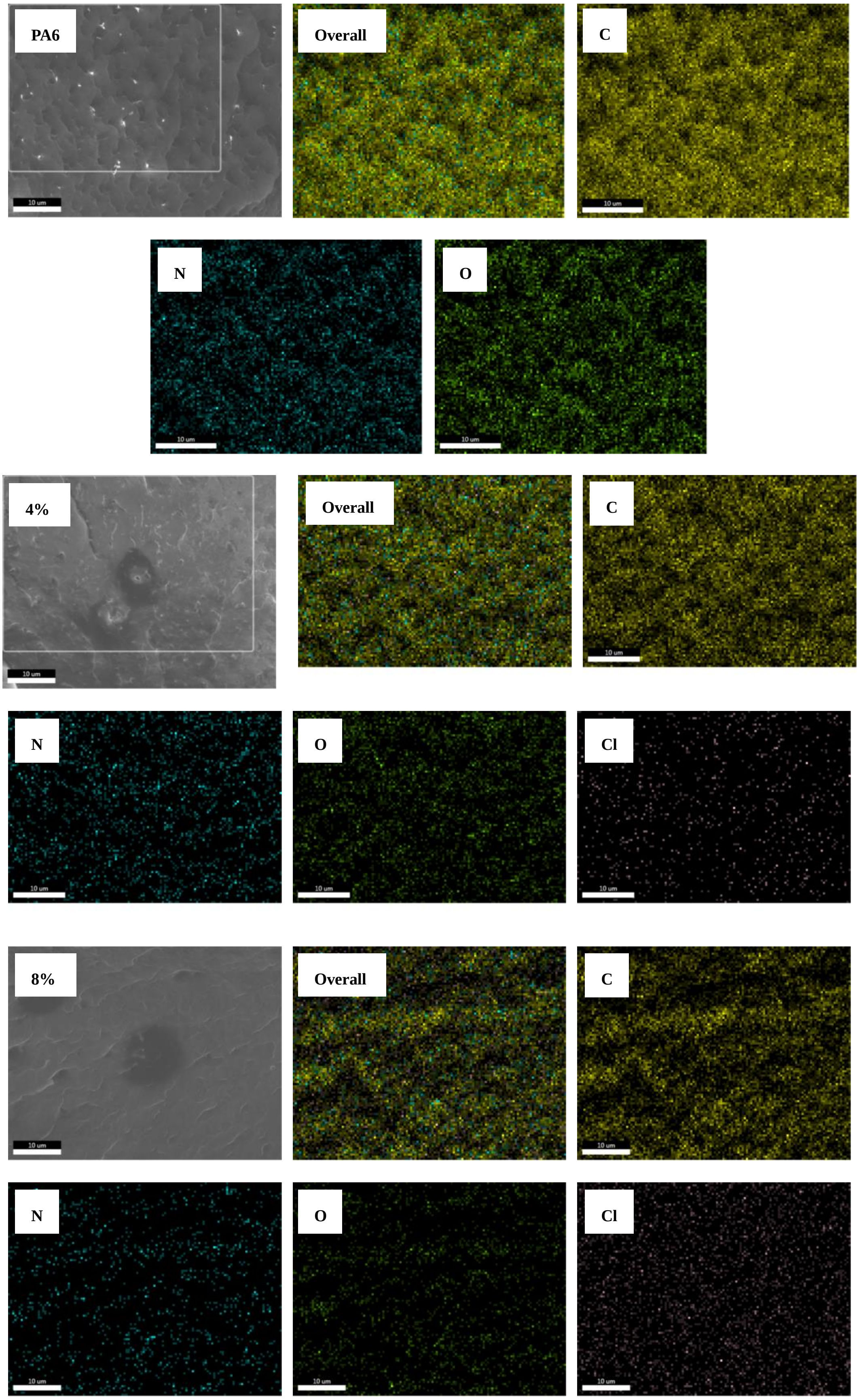

Figure S1. Elemental mapping distribution of PA6, PA6/VBIM, 4wt% blend (labelled as 4%) and PA6/VBIM, 8wt% blend (labelled as 8%).

Elemental mapping distribution of PA6 and PA6 blends with relative high VBIM content (4-8 wt %) is given in Figure S1. It provided an effective and visualized way to observe the dispersion of VBIM in PA6 matrix. It is obvious that only C, N, O were detected in neat PA6. While in PA6/VBIM blends, the characteristic Cl of VBIM was observed except of the inherent elements of C, N, O in PA6 matrix. This is a strong evidence that VBIM was incorporated into PA6 matrix successfully. Besides, it can be seen from the images of 4% and 8% that Cl distributes uniformly in the matrix, indicating a good miscibility of VBIM in PA6 matrix, which confirmed the results of SEM observation convincingly. In addition, the amount of Cl in PA6/VBIM, 8 wt % blend is more than that in PA6/VBIM, 4 wt % blend, which is also consistent with the incorporated amount of VBIM.

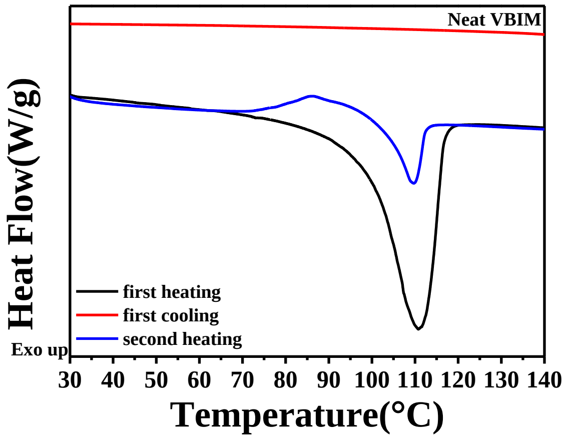

Figure S2. The DSC curves of neat VBIM.

The crystallization and melting behaviors of VBIM have been investigated by DSC measurement, the dried IL was heated to 150 °C and held for 5 min to erase thermal history, then cooled down to 25 °C, followed by heating again to 150 °C. The curves are shown in Figure S2. It's obvious that the IL was crystallized at room temperature so that there is an obvious melting peak at 111 °C during first heating process. No evident crystallization peak was observed in the cooling track at whole tested temperature range. But a broad cold crystallization peak was detected at about 86 °C during second heating process, indicating a poor crystallization capacity and a slow crystallization rate. The melting temperature on second heating track was appeared to be 110 °C, which is very close to the first melting temperature (111 °C), indicating the stability of IL when heating to at least 150 °C.

Table S1. Basic tensile properties of the neat PA6 and PA6/VBIM blends.

Sample	Yield Strength (MPa)	Elongation at break (%)	Modulus (MPa)	Strength at break (MPa)
PA6	63.0±1.4	347.0±69.7	1258.7±27.6	77.1±9.2
PA6/VBIM,0.25%	67.8(108%)±1.9	333.7±46.0	1718.3±23.8	72.1±3.1
PA6/VBIM, 0.5%	69.2(110%)±14.9	312.7±31.4	1522.7±65.2	81.0±53.0
PA6/VBIM, 1%	67.9(108%)±0.6	288.5±20.5	1441.9±98.9	79.1±0.6
PA6/VBIM, 2%	65.6(104%)±0.2	310.5±11.3	1444.2±30.2	79.3±0.3
PA6/VBIM, 4%	63.9(101%)±1.0	304.0±32.8	1234.7±13.6	77.9±4.0
PA6/VBIM, 8%	48.1(76%)±1.1	386.4±34.8	803.1±20.0	73.3±3.6

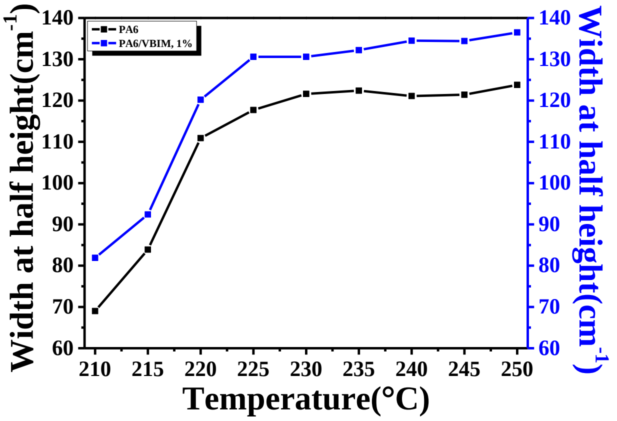

Figure S3. The plot of N-H width at half height in IR as a function of temperature.

The FTIR spectra of PA6 and PA6/VBIM blends with different VBIM content were recorded respectively at room temperature. As is shown in Figure S4, the characteristic peak of neat PA6 at 3300 cm^{-1} represents the N-H stretching vibration. It is sensitive to the strength of intermolecular interactions. The small shoulder at about 3210 cm^{-1} is due to the Fermi resonance of the N-H stretching vibration. The band at 3079 cm^{-1} is contributed to the combination band of the amide I and amide II modes. The peaks at 2935 and 2864 cm^{-1} are respectively assigned to the asymmetric and symmetric stretching of CH_2. The two bands at 1639 and 1546 cm^{-1} are amide I and amide II bands. For neat VBIM, The multiple peaks at 3190~2640 cm^{-1}and the strong peak at 1175 cm^{-1} are assigned to the imidazolium group, and peaks locate at 891~1000 cm^{-1} and 1651 cm^{-1} are characteristic peaks of the vinyl group. But, unfortunately, no obvious peaks of VBIM were observed in PA6/VBIM blends because the peaks of VBIM were overlapped by those of PA6. Besides, no apparent peak shift of PA6 occurred with the combination of VBIM. Take the characteristic peak of C=O and N-H for example, the peaks located at 3300 and 1639 cm^{-1} in neat PA6 respectively, while the C=O and N-H peaks of PA6/VBIM, 0.5-4 wt % emerged at almost the same position with neat PA6, indicating that 0.5-4 wt % content of VBIM show little impact on the inherent properties of functional groups, particularly the hydrogen bond between amide groups in PA6 molecular chains. That is to say, a certain amount of VBIM (0.5-4 wt %) do not break the original hydrogen bond in PA6 matrix. It is suggested that the changes in peak position, peak strength and peak shape of FTIR spectrum are all noteworthy to discern small structure transformation. We found that neither of the characteristic peaks of N-H nor that of C=O is single Gaussian curve both in neat PA6 and VBIM incorporated PA6 blends, and the shape of the peaks changed markedly when VBIM was added.

More interestingly, slight shifts were observed in PA6/VBIM, 8 wt % blend. To be specific, the peak of N-H shift from 3300 to 3297 cm^{-1} and the peak position of C=O locates at 1638 cm^{-1} which is 1 cm^{-1} lower than that in neat PA6. Both the two peaks shift to lower wavenumber signs that there are stronger interactions generated as 8wt% content of VBIM added to PA6 matrix. We believed that the corporation of VBIM tends to form new hydrogen bond with free amide groups. Specifically, the imidazolium cations, with active hydrogen as hydrogen bond acceptor, would possibly preferred to coordinate with the "free" carbonyl oxygen atom while the chloride anions may form new hydrogen bonds with "free" amino groups. The electron withdrawing effect of the imidazolium cation on C=O group lead to a decrement of C=O strength so that the coordination with imidazolium cation shift the amide I band to a lower frequency. The neighboring N-H groups should also have a longer and weaker bond because of the electron transfer from nitrogen atom to the imidazolium cation, but the electron deficiency property of this kind of N-H make it a better acceptor for another powerful electron

donator, the chloride anion. The electron compensation of chloride anion on the N-H group also shift the peak to lower wavenumber.

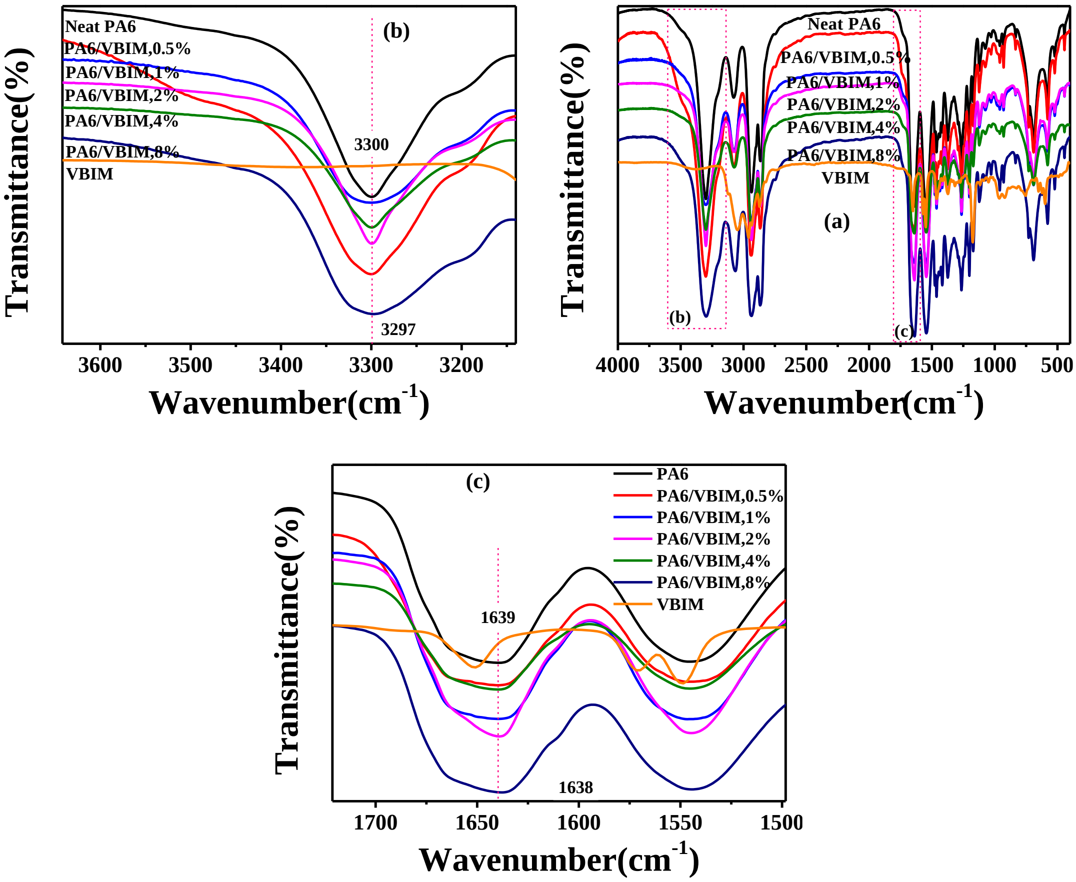

Figure S4. FTIR spectra of: (a) neat PA6, VBIM and PA6/VBIM blends; (b) zoomed area of N-H stretching band; (c) zoomed area of amide I band.
